# AutoCCS: automated collision cross-section calculation software for ion mobility spectrometry–mass spectrometry

**DOI:** 10.1093/bioinformatics/btab429

**Published:** 2021-06-19

**Authors:** Joon-Yong Lee, Aivett Bilbao, Christopher R Conant, Kent J Bloodsworth, Daniel J Orton, Mowei Zhou, Jesse W Wilson, Xueyun Zheng, Ian K Webb, Ailin Li, Kim K Hixson, John C Fjeldsted, Yehia M Ibrahim, Samuel H Payne, Christer Jansson, Richard D Smith, Thomas O Metz

**Affiliations:** Earth and Biological Sciences Directorate, Pacific Northwest National Laboratory, Richland, WA 99352, USA; Earth and Biological Sciences Directorate, Pacific Northwest National Laboratory, Richland, WA 99352, USA; Earth and Biological Sciences Directorate, Pacific Northwest National Laboratory, Richland, WA 99352, USA; Earth and Biological Sciences Directorate, Pacific Northwest National Laboratory, Richland, WA 99352, USA; Earth and Biological Sciences Directorate, Pacific Northwest National Laboratory, Richland, WA 99352, USA; Earth and Biological Sciences Directorate, Pacific Northwest National Laboratory, Richland, WA 99352, USA; Earth and Biological Sciences Directorate, Pacific Northwest National Laboratory, Richland, WA 99352, USA; Earth and Biological Sciences Directorate, Pacific Northwest National Laboratory, Richland, WA 99352, USA; Department of Chemistry & Chemical Biology, Indiana University–Purdue University Indianapolis, Indianapolis, IN 46202, USA; Earth and Biological Sciences Directorate, Pacific Northwest National Laboratory, Richland, WA 99352, USA; Earth and Biological Sciences Directorate, Pacific Northwest National Laboratory, Richland, WA 99352, USA; Agilent Technologies, Santa Clara, CA 95051, USA; Earth and Biological Sciences Directorate, Pacific Northwest National Laboratory, Richland, WA 99352, USA; Department of Biology, Brigham Young University, Provo, UT 84602, USA; Earth and Biological Sciences Directorate, Pacific Northwest National Laboratory, Richland, WA 99352, USA; Earth and Biological Sciences Directorate, Pacific Northwest National Laboratory, Richland, WA 99352, USA; Earth and Biological Sciences Directorate, Pacific Northwest National Laboratory, Richland, WA 99352, USA

## Abstract

**Motivation:**

Ion mobility spectrometry (IMS) separations are increasingly used in conjunction with mass spectrometry (MS) for separation and characterization of ionized molecular species. Information obtained from IMS measurements includes the ion’s collision cross section (CCS), which reflects its size and structure and constitutes a descriptor for distinguishing similar species in mixtures that cannot be separated using conventional approaches. Incorporating CCS into MS-based workflows can improve the specificity and confidence of molecular identification. At present, there is no automated, open-source pipeline for determining CCS of analyte ions in both targeted and untargeted fashion, and intensive user-assisted processing with vendor software and manual evaluation is often required.

**Results:**

We present AutoCCS, an open-source software to rapidly determine CCS values from IMS-MS measurements. We conducted various IMS experiments in different formats to demonstrate the flexibility of AutoCCS for automated CCS calculation: (i) stepped-field methods for drift tube-based IMS (DTIMS), (ii) single-field methods for DTIMS (supporting two calibration methods: a standard and a new enhanced method) and (iii) linear calibration for Bruker timsTOF and non-linear calibration methods for traveling wave based-IMS in Waters Synapt and Structures for Lossless Ion Manipulations. We demonstrated that AutoCCS offers an accurate and reproducible determination of CCS for both standard and unknown analyte ions in various IMS-MS platforms, IMS-field methods, ionization modes and collision gases, without requiring manual processing.

**Availability and implementation:**

https://github.com/PNNL-Comp-Mass-Spec/AutoCCS.

**Supplementary information:**

Supplementary data are available at *Bioinformatics* online. Demo datasets are publicly available at MassIVE (Dataset ID: MSV000085979).

## 1 Introduction

Ion mobility spectrometry (IMS) coupled with mass spectrometry (MS) is increasingly utilized in studies of biomolecules as it offers highly reproducible and extremely fast separations (milliseconds to seconds). IMS separates molecules in the gas phase based on their collision cross sections (CCS) with a neutral buffer gas, a property that represents the three-dimensional structure of the corresponding ion. IMS-MS provides high throughput, two-dimensional information (i.e. mass and mobility) enabling the identification and quantification of metabolites with greater confidence and effectiveness than approaches based on MS alone.

Automated data processing tools are increasingly critical as instrumentation, data density and acquisition speed advance. Unfortunately, processing the large amounts of data generated in large-scale IMS analyses is still a bottleneck (Stow *et al.*, 2017; Zheng *et al.*, 2017). We previously developed PIXiE, which is a software for automated arrival time extraction and CCS calculation of standard analytes measured using the ‘stepped-field’ method in drift tube-based IMS (DTIMS)-MS instruments (Ma *et al.*, 2017).

Here, we present a new IMS-MS software tool, AutoCCS, capable of calculating CCS from stepped- (i.e. multi-) field data and single-field analyses of unknowns where CCS determination is based on calibration against well-characterized ions measured under the same conditions. In contrast to PIXiE, which only performs a targeted extraction of predefined ions for stepped-field IMS-MS measurements, AutoCCS can utilize features detected from single-field IMS-MS measurements in an untargeted fashion to calculate CCS values for all measurable analytes and their various conformers. These functionalities enable CCS determination for unknown molecules, as well as for experiments performed on traveling wave based-IMS (TWIMS), including Waters Synapt and Structures for Lossless Ion Manipulations (SLIM) IMS devices (Ibrahim *et al.*, 2017). For single-field methods, AutoCCS supports internal or external calibration (with an arbitrary or configurable list of reference ions) and an ‘enhanced’ CCS calibration mode with external calibration that corrects for temperature and pressure variations that may be present in sample runs that are temporally distant from the calibrant run. Also, AutoCCS performs non-linear regression analysis with either a polynomial or linearized power function for TWIMS data. For use with in-house SLIM IMS-MS systems, we modified the typical non-regression methods by incorporating the accumulation time for the arrival time adjustment.

We demonstrate AutoCCS with data collected in various IMS systems and methods: DTIMS-MS, RapidFire-DTIMS-MS, TWIMS-MS, trapped IMS (TIMS)-MS and SLIM IMS-MS, for both authentic standard reference compounds and complex samples in various conditions (e.g. buffer gas and ionization mode).

## 2 Materials and methods

### 2.1 Overview of AutoCCS

AutoCCS is a freely available command-line tool to determine CCS values from various IMS-MS platforms and data acquisition modes. It is written in Python for greater accessibility and is complementary to other data processing layers, such as raw MS-data preprocessing [i.e. PNNL-PreProcessor (Bilbao, 2020)] and feature finding. The main input is the list of IMS features (i.e. tables of mass and arrival time), which can be generated with the user’s preferred software (Agilent proprietary Mass Profiler .cef and open-source MZmine .csv formats are currently supported). 

AutoCCS allows users to change configurations as needed to calculate CCS in various use cases. Configuration parameters (e.g. Supplementary Fig. S1) and command-line options that users can control can be found in Supplementary Tables S1 and S2, respectively.

**Fig. 1.**
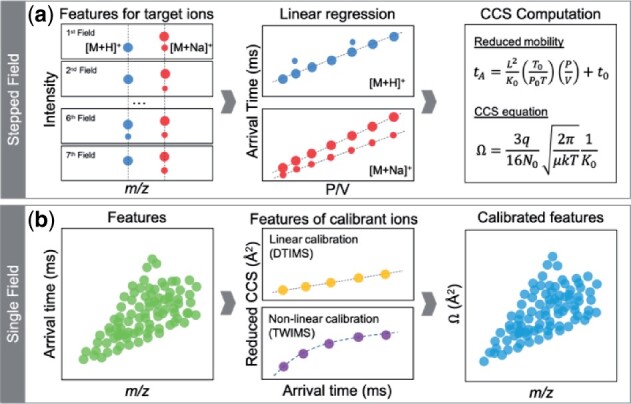
Overall workflow of AutoCCS for different types of IMS-MS experiments. (**a**) Stepped-field (also known as multi-field) example with IMS features found in each field for a target molecule and two configured adducts (protonated in blue and sodiated in red). The features from each field are grouped to fit a linear regression model, from which the slope derives the mobility (*k*_0_) of the ions and is used in the fundamental ion mobility equation (Revercomb and Mason, 1975) to determine the CCS. The AutoCCS comprehensive output includes all detected conformers and various metrics to help researchers evaluate the quality and select plausible CCS values. For the sodiated adduct shown, two high-quality conformers were found with high and medium intensity features detected in all fields. Only one high-quality conformer was detected for the protonated adduct with high intensity (the smaller intensity conformer was detected in only two fields). (**b**) Single-field example with all (unknown) detected features shown in green before calibration. Features detected for the calibrant ions with known CCS (internal or external measured under the same conditions) are used to build the calibration curve and calculate CCS for the unknown features, which are shown in blue after calibration. Based on the calibrants, linear regression is performed for DTIMS and timsTOF (yellow features on top panel) and non-linear regression (with polynomial or linearized power functions) is utilized for TWIMS (purple features on bottom panel)

For the stepped-field DTIMS method (Stow *et al.*, 2017), AutoCCS allows users to automatically determine CCS values for targeted ions by performing linear regression using various fields(Fig. 1a). For the single-field DTIMS method (Kurulugama *et al.*, 2015), AutoCCS supports two modes: the standard method and also an ‘enhanced’ calibration method to improve CCS determination in high-throughput experiments by accounting for temperature and pressure variations between calibrant and complex sample analyses (Fig. 1b). For TWIMS methods, AutoCCS offers a polynomial function and linearized power function to apply to calibration curves.

### 2.2 Stepped-field DTIMS

A traditional, standardized stepped-field DTIMS method provides straightforward and highly reproducible accurate CCS determination, with very low relative standard deviations, even among different laboratories (Stow *et al.*, 2017). The stepped-field method employed in DTIMS takes advantage of the linear relationship of arrival time ($t_{A}$) and reduced mobility ($K_{0}$), as follows:

(1) $$t_{A}=\;\frac{L^{2}}{K_{0}}\left(\frac{273.15}{760T}\right)\left(\frac{P}{V}\right)+\;t_{0}.$$

using the length of the drift tube (L), temperature (T), drift gas pressure (P), drift voltage (V) and $t_{0}$ which encompasses time spent outside of the drift cell. Based on this linear regression analysis, the molecular CCS values can be directly computed as below (Revercomb and Mason, 1975):

(2) $$\Omega =\frac{3q}{16N_{0}}\sqrt{\frac{2\pi }{\mu kT}}\frac{760}{P}\frac{T}{273.15}\frac{t_{A}E}{L}=\frac{3q}{16N_{0}}\sqrt{\frac{2\pi }{\mu kT}}\frac{1}{K_{0}}\;$$

where q=ze is the ionic charge (z: charge state of the ion, e: unit electronic charge), $N_{0}$ is the number density of the drift gas, μ is the reduced mass of the ion, k is the Boltzmann constant and E(=V/L) is the electric field.

AutoCCS allows users to automatically search for multiple adduct ions. A list of target ions with neutral exact masses (e.g. Supplementary Table S3) and metadata files with IMS acquisition conditions (e.g. electric fields, pressures and temperatures which are exported using the PNNL-PreProcessor) are also required. Based on the exact mass and configured adducts, the target ions are generated and selected from the measured mass-to-charge (*m/z*) ratio of the given IMS features and *m/z* tolerance as defined by users. For each combination of the selected features (i.e. arrival time) in all *n* fields, AutoCCS performs linear regression analysis between $\{P_{1}/V_{1},\;P_{2}/V_{2},\;\ldots ,P_{n}/V_{n}\}\;$and $\left\{t_{a}^{1i},t_{a}^{2j},\ldots ,t_{a}^{nk}\right\}$ where $t_{a}^{ni}$ indicates the arrival time of the ith features in the nth field, and $P_{n}$ and $V_{n}$ represents the pressure and temperature in the nth field, respectively. Among all combinations of selected features, AutoCCS identifies the best fit regressors by the $R^{2}$ threshold value (e.g. $R^{2}\geq 0.99$ by default), which users can customize. To reduce the number of combinations, users can limit the number of selected features in each field based on peak intensity ranks. See the details about the user parameters in Supplementary Tables S1 and S2.

AutoCCS delivers the following output files for the stepped-field method analysis: (i) a tab-delimited text file containing calculated CCS values of target ions found for each standard compound and all information about the experimental features used (e.g. arrival time, intensity, mass error and linear regression analysis in Supplementary Table S4), (ii) plots of linear regressors and the selected features (Supplementary Fig. S2), (iii) plots of the distributions of feature intensities (Supplementary Fig. S3) and (iv) plots of the metadata (temperatures, pressures and voltages in drift tubes) in all frames (Supplementary Fig. S4).

### 2.3 Single-field DTIMS

As an alternative to the stepped-field method, a single-field method has been widely accepted as more practical for estimating the CCS values of detected ions in complex samples, particularly when liquid chromatography and similar front-end separation methods are coupled with DTIMS. Contrary to the stepped-field method that depends on multiple instrument parameters, the single-field DTIMS method (Kurulugama et al., 2015) is based on a linear regression using known CCS (from literature or stepped-field experiments) of calibrant species. An example of the list of calibrant ions is in Supplementary Table S5. This calibration curve allows CCS determination of other analytes from their arrival times measured under the same IMS conditions as the calibrants. As such, for the single-field CCS determination a target list of analyte ions is not required and AutoCCS processes data in an untargeted fashion by applying the calibration function to all detected features.

### 2.4 Enhanced single-field DTIMS

In a typical single-field experiment, there is an assumption that temperature and pressure will not change between the calibrant and actual sample analyses (i.e. using ‘external’ calibration). In practice, the analysis of calibrants is usually performed once or twice a day in order to maintain the data acquisition throughput for the analysis of real samples. However, the instrument conditions (e.g. temperature and pressure) may vary enough to affect the CCS determination if there is a considerable time gap (e.g. few hours) between the calibrant and real sample analyses or if the ambient laboratory conditions changed significantly.

To reduce the undesired effects of both temperature and pressure variations between sample runs and to improve accuracy using external calibration, we implemented an enhanced single-field method with an adjusted CCS ($\Omega^{\prime \prime }$) derived by additionally accounting for temperature (T) and pressure (P) to the reduced CCS ($\Omega^{'}$), used in a standard method, as shown in Equation (3):

(3) $$\Omega^{'}=\frac{1}{z}\sqrt{\frac{m_{I}}{m_{I}+m_{B}}}\Omega \;\mathrm{and}\;\Omega^{\prime \prime }=\frac{P}{\sqrt{T}}\Omega^{\prime }=\frac{P}{z\sqrt{T}}\sqrt{\frac{m_{I}}{m_{I}+m_{B}}}\Omega \;$$

where z, $m_{I}$, $m_{B}$ and Ω denote the ion charge state, the ion mass, the buffer gas mass and the known CCS value, respectively. Combining Equations (1) and (2), a linear calibration function can be derived as follows (Marchand *et al.*, 2017; Stow *et al.*, 2017):

(4) $$t_{A}=\frac{\sqrt{\mu }}{z}\Omega \frac{P}{\sqrt{T}}\cdot \frac{{16N_{0}L}^{2}}{3e}\cdot \sqrt{\frac{k}{2\pi }}\cdot \frac{273.15}{760V}+\;t_{0}.$$

Pressure and temperature terms were incorporated into the reduced CCS in the enhanced method whereas empirical correlation with $P/\sqrt{T}$ has been ignored as a constant in the conventional method. AutoCCS provides an option to select whether to use this enhanced version or to use the default one to characterize the calibration curve.

For single-field methods, AutoCCS visualizes the calibration curves, which facilitates visual inspection of the curve fitting result and its quality (Supplementary Fig. S5) and easy verification of selected ions and the regression models.

### 2.5 Calibration methods for TWIMS

For TWIMS instrumentation, such as for SLIM devices, CCS cannot be calculated directly using the mathematical function provided by the fundamental low-field ion mobility equation (Revercomb and Mason, 1975) due to the variable electric field used in TWIMS (Gabelica *et al.*, 2019; Lanucara *et al.*, 2014; Ruotolo *et al.*, 2008). Unlike the single-field method in DTIMS, a non-linear relationship needs to be accounted for effective calibration in TWIMS. For this purpose, AutoCCS performs either a polynomial (Bush *et al.*, 2012) or power (Gabelica *et al.*, 2019; Ruotolo *et al.*, 2008) regression analysis using the arrival time $t_{A}$ and known CCS values of reference compounds to create a calibration function for determination of TWIMS CCS.

For polynomial regression, data for each calibrant run is fit using the Equation (5):

(5) $$\Omega^{'}=x_{0}+{x_{1}t}_{A}^{'}+x_{2}{t^{'}}_{A}^{2}+\cdots +x_{D}{t^{'}}_{A}^{D}\;\mathrm{and}\;t_{A}^{'}=t_{A}-t_{\mathrm{acc}}\;$$

where D denotes the degree of the polynomial function that users choose, $t_{\mathrm{acc}}$ represents the ion accumulation time in the SLIM system and $\Omega^{'}$ represents the reduced CCS. For example, if D=2 or 3, it becomes a quadratic function for the binomial regression or a cubic function for the trinomial regression, respectively.

In the power regression, the calibrant arrival times were corrected by Equation (6):

(6) $$t_{A}^{'}=t_{A}-\frac{C\sqrt{\frac{m}{z}}}{1000}-t_{\mathrm{acc}}\;$$

where C is a constant coefficient designated as the EDC (Enhanced Duty Cycle) delay coefficient for the Synapt Q-IM-o-ToF instrument (Ruotolo *et al.*, 2008), but was set to 0 for the SLIM system. Then the linear relationship between corrected arrival time ($t_{A}^{'}$) and the reduced CCS values ($\Omega^{'}$) is fit to the linearized power function as follows:

(7) $$\ln\Omega^{'}=X\times \ln t_{A}^{'}+\ln Y\;$$

where *X* and *Y* denote the coefficients of the power function. From this regression analysis, we calculate the new corrected arrival time ($t_{A}^{''}$) from Equation (8):

(8) $$t_{A}^{''}={t_{A}^{'}}^{X}\times z\times \sqrt{1/\mu }\;$$

where *z* and *μ* denote a charge state and a reduced mass of the ion, respectively. Finally, $t_{A}^{''}$ can be translated to the known CCS values (Ω) by fitting a linear regressor as follows:

(9) $$\Omega =a\times t_{A}^{''}+b.\;$$

### 2.6 Sample preparation

Agilent ESI-L low concentration tuning mix solution (Agilent Santa Clara, CA; https://www.agilent.com/cs/library/certificateofanalysis/G1969-85000cofa872022-U-LB86189.pdf) was used without further purification, hereafter referred as tune-mix. Tetraalkyl ammonium (TAA) salts were purchased from Sigma-Aldrich (Milwaukee, WI USA) and used without any further purification. The TAA salts, consisting of tetraethylammonium through tetraoctylammonium, were prepared as a mixture to a final concentration of 1 µM in 50/50 water/methanol with 0.5% acetic acid (*v*/*v*). Rotenone was spiked into a complex background of 3553 plant leaf extracts at 0.001 mg/ml. Briefly, leaf samples were harvested from a field located at the University of California Agriculture & Natural Resources (UC-ANR) Kearney Agricultural Research & Extension Center (KARE) in Parlier, CA for a large-scale plant study to assay the natural diversity of *Sorghum bicolor*. The leaves were placed into 50-ml conical tubes and frozen with liquid nitrogen in the field and then lyophilized. Dried samples were pulverized and ground. 60 mg aliquots were measured out of each leaf sample for metabolite extraction. 1 ml of 80:20 MeOH: H2O was added and samples were centrifuged. The supernatant was removed (∼800 µl) and further aliquoted into conical bottom 96 well plates, analyzed via solid-phase extraction with IMS-MS (SPE-IMS-MS) (Zhang *et al.*, 2016).

The IROA standards (Adenine, d-(+)-Trehalose and Pterin) were purchased from IROA Technologies (Sea Girt, NJ, USA), and all reagents were purchased from Thermo Fisher Scientific (Waltham, MA, USA). Standards were supplied as 5 µg dried weight and reconstituted using a combination of 0.1% acetic acid, 79.9% methanol and 19.9% water. Stock solutions of the standards were prepared at either 100 or 500 µM concentration and then further diluted to a concentration of 10 µM prior to analysis.

### 2.7 Data acquisition

#### 2.7.1 Stepped-field DTIMS-MS measurements

For the experiments with nitrogen, the Agilent tune-mix solution was analyzed by direct infusion using an Agilent jet stream orthogonal electrospray ionization source maintained at the following parameters: nitrogen sheath gas, sheath gas temperature, drying gas, drying gas temperature, nozzle voltage and inlet capillary voltage of at 8 l/min, 275°C, 3 l/min, 325°C, 2 kV and 4 kV respectively. Data were acquired on an Agilent 6560 Ion Mobility QTOF MS system (Agilent Technologies, Santa Clara, CA) using a stepped-field method and the drift potential was varied between 850 and 1450 V by 100 V increments, and each drift potential was acquired for 30 s.

For the IROA standards and TAA mixture experiments with helium, a customized flow injection system (Orton *et al.*, 2018) was coupled to an in-house built IMS-MS instrument that combines a 1 m drift tube with an Agilent 6538 QTOF MS (Ibrahim *et al.*, 2015) and is comparable to the commercial Agilent 6560 system but providing increased IMS resolution. For these experiments, the drift potential was varied between 677 and 1198 V by 87 V increments, and each drift potential was acquired for 44 s. The IMS was pressurized with ultrahigh purity helium, and the trapping funnel pressure and drift tube pressure were maintained at 3.8 and 4.0 Torr, respectively.

#### 2.7.2 SLIM IMS measurements

The SLIM TWIMS-MS platform used for these experiments has been described in detail elsewhere (Wojcik *et al.*, 2019) and is comparable to an instrument that will soon be commercially available (https://mobilionsystems.com). The Agilent tune-mix, TAA mixture and IROA standards were infused by a customized flow injection system (Orton *et al.*, 2018) at 300 nl/min for nano-electrospray ionization at 3 kV into an inlet capillary heated to 130°C. Ions pass through an ion funnel (3.60 Torr, helium buffer gas) before entering the SLIM module (3.80 Torr, He buffer gas). As described previously, ions were accumulated in the SLIM module at an interface 9 m into the 13.5 m ion path (Deng *et al.*, 2017). For these experiments, an accumulation period of 1000 ms was performed prior to separation in the remaining 4.5 m at a TW amplitude of 12 Vpp and TW speed of 180 m/s. An Agilent 6224 TOF mass spectrometer (Agilent Technologies, Santa Clara, CA) was used for all experiments. All SLIM TWIMS-MS data was acquired using home-built software.

#### 2.7.3 Synapt IMS measurements

The Agilent tune-mix solution was infused into a Waters Synapt G2s-i mass spectrometer using a static nanoelectrospray source. A borosilicate glass emitter (in house pulled using Sutter Instrument, model P-1000) containing the sample was in contact with a platinum wire, applied with 1.3 kV for electrospray. Source temperature was 150°C and sampling cone at 150 V. No source gas was used. Instrument tune parameters were at default values. Trap (argon), helium cell and IMS (nitrogen) gas flow was 2, 180 and 90 ml/min, respectively. Traveling wave (default) settings were 313 m/s 4 V trap, 650 m/s 40 V IMS and 175 m/s 4 V transfer. Spectra were averaged over 1.6 min, and the IMS peak list was generated using default peak detection settings in DriftScope v2.4 and manually formatted as MZmine csv output. EDC delay coefficient was 1.45.

#### 2.7.4 Bruker timsTOF data

Agilent tune-mix data were kindly provided by Bruker Corporation. Data were acquired on a Bruker timsTOF Pro™ instrument equipped with trapped ion mobility coupled to a QTOF MS. Ions were generated in positive electrospray ionization mode using a scan range of 100–1600 *m/z*. Tune mix features were extracted using MetaboScape and IMS scan numbers were used as a substitute for drift times for CCS calibration.

#### 2.7.5 Single-field DTIMS-MS measurements with RapidFire

Plant extracts were analyzed by SPE-IMS-MS using a RapidFire 365 (Agilent Technologies, Santa Clara, CA) coupled with an Agilent 6560 Ion Mobility QTOF MS system (Agilent Technologies, Santa Clara, CA). Samples were loaded onto Graphitic Carbon and C18 SPE cartridges. The IMS was pressurized with ultrahigh purity nitrogen. All data were acquired in positive electrospray mode with a mass range of *m/z* 50–1700. A total of 7106 metabolite IMS runs were acquired. Agilent tune-mix was analyzed daily for single-field CCS calibration (28 calibrant runs).

### 2.8 IMS data pre-processing and feature finding

The Unified Ion Mobility Frame (UIMF) file format (https://github.com/PNNL-Comp-Mass-Spec/UIMF-Library) was used for in-house IMS/SLIM platforms to store both the raw data and the metadata associated with an IMS-MS experiment in a single cross platform file. Raw MS files (MassHunter ‘.d’ or UIMF format) were pre-processed in batch mode using the PNNL-PreProcessor v2020.07.24 (https://omics.pnl.gov/software/pnnl-preprocessor) to apply smoothing and generate new raw files with all frames (ion mobility separations) summed into a single frame and export frame metadata text files containing the IMS acquisition conditions (e.g. electric fields, pressure and temperature). For the stepped-field method, the option to split the file by field was used and a new raw file was generated for each field. For RapidFire, the first and last four frames were excluded to remove chemical noise and background ions. Ion mobility smoothing using 3 points and a minimum intensity threshold of 30 counts were applied for DTIM files, and 15 points and a minimum intensity threshold of 1 count for SLIM files. For Agilent and in-house SLIM data processing, we employed the PNNL-PreProcessor in the pipeline. However, any other tools can be applied for feature finding (i.e. identifying peaks with *m/z* and drift time). The PreProcessor was mandatory for the stepped-field method, since it was the only tool with the functionality to export the IMS instrument parameters needed for the stepped-field and the enhanced single-field CCS calculations.

Single-frame files were converted to mzML using ProteoWizard v3.0.19228 64-bit (Kessner *et al.*, 2008), arrival time was used as a substitute for retention time to generate ‘LC-MS-like’ files and feature detection was performed in batch mode using MZmine 2 v2.41.2 (Pluskal *et al.*, 2010) with the steps: mzML raw data import, mass detector ‘Wavelet transform’, chromatogram builder, chromatogram deconvolution (‘Local minimum search’ for DTIM and ‘Noise amplitude’ for SLIM) and CSV data export. Supplementary Figure S6 describes the complete procedures for IMS data pre-processing and feature finding from the raw data files.

### 2.9 CCS calculation using IM-MS browser

The Agilent MassHunter IM-MS Browser v10.0 was used for manual CCS determination. For single-field runs, the corresponding infusion run of the tune-mix was used for CCS calibration followed by IMFE feature finding and exporting of features as csv. For stepped-field calculation of each run, all frames from the 4th field were selected and summed and then each target adduct ion was selected by boxing the first two isotopic peaks to run the CCS calculation and copy-and-paste the results in MS-Excel.

## 3 Results

### 3.1 Tune-mix sample runs in different IMS platforms and modes

To evaluate the performance of AutoCCS, we conducted five infusion experiments with tune-mix sample runs in positive electrospray mode: (i) stepped-field DTIMS-MS utilizing seven electric fields, (ii) single-field DTIMS-MS, (iii) TWIMS analysis on a SLIM and (iv) Waters Synapt and (v) trapped IMS data from a Bruker timsTOF. Table 1 shows that the reproducibility of the CCS determinations of the ions from the Agilent tune-mix sample was excellent across these IMS systems and that AutoCCS was effective for all IMS methods, automatically calculating CCS values with acceptable differences compared to manually determined CCS using vendor software.

**Table 1.** CCS values calculated for Agilent tune-mix samples measured in positive electrospray mode in nitrogen drift gas with four different IMS platforms

Type	Tune-mix calibrants	Adduct Ions *m/z*	AutoCCS CCS (Å^2^)	^1^CCS_Browser_ (Å^2^)	^1^ Δ CCS_Browser_ (%)	^2^CCS_PNNL_ (Å^2^)	^2^ Δ CCS_PNNL_ (%)	^3^CCS_McLean_ (Å^2^)	^3^ Δ CCS_McLean_ (%)
Stepped-field DTIMS-MS	TuneMix321	322.0481	153.6123	153.9	0.19	153.7	0.06	153.4(1)	0.14(1)
								153.5(6)	0.07(6)
	TuneMix621	622.0290	202.9291	203.5	0.28	203.0	0.03	202.8(6)	0.06(6)
								203(8)	0.03(8)
	TuneMix921	922.0098	244.9022	244.4	0.21	243.6	0.53	243.9(1)	0.41(1)
								243.5(6)	0.58(6)
	TuneMix1220	1221.9906	283.1977	282.9	0.11	282.2	0.35	283.1(1)	0.03(1)
								281.8(6)	0.50(6)
	TuneMix1520	1521.9715	317.0891	317.8	0.22	317.0	0.03	318.9(1)	0.57(1)
								316.5(6)	0.19(6)
Single-field DTIMS-MS	TuneMix321	322.0481	153.9290	153.8	0.08	153.7	0.15	153.4(1)	0.34(1)
								153.5(6)	0.28(6)
	TuneMix621	622.0290	203.0138	203.0	0.01	203.0	0.01	202.8(6)	0.11(6)
								203(8)	0.01(8)
	TuneMix921	922.0098	243.4948	243.6	0.04	243.6	0.04	243.9(1)	0.17(1)
								243.5(6)	0.00(6)
	TuneMix1220	1221.9906	281.7040	282.1	0.14	282.2	0.18	283.1(1) 281.8(6)	0.49(1) 0.03(6)
	TuneMix1520	1521.9715	317.4272	317.0	0.13	317	0.13	318.9(1)	0.46(1)
								316.5(6)	0.29(6)
TWIMS-MS (SLIM)	TuneMix621	622.0290	203.1121	—	—	203	0.06	202.8(6)	0.15(6)
								203(8)	0.06(8)
	TuneMix921	922.0098	243.2938	—	—	243.6	0.13	243.9(1)	0.25(1)
								243.5(6)	0.08(6)
	TuneMix1220	1221.9906	282.4849	—	—	282.2	0.10	283.1(1)	0.22(1)
								281.8(6)	0.24(6)
	TuneMix1520	1521.9715	316.9095	—	—	317	0.03	318.9(1)	0.62(1)
								316.5(6)	0.13(6)
TWIMS-MS (SYNAPT)	TuneMix321	322.0481	154.1847	—	—	153.7	0.32	153.4(1)	0.51(1)
								153.5(6)	0.45(6)
	TuneMix621	622.0290	202.9855	—	—	203	0.01	202.8(6)	0.09(6)
								203(8)	0.01(8)
	TuneMix921	922.0098	242.8969	—	—	243.6	0.29	243.9(1)	0.41(1)
								243.5(6)	0.25(6)
	TuneMix1220	1221.9906	281.7227	—	—	282.2	0.17	283.1(1)	0.49(1)
								281.8(6)	0.03(6)
	TuneMix1520	1521.9715	317.4757	—	—	317	0.15	318.9(1)	0.45(1)
								316.5(6)	0.31(6)
TIMS-MS (timsTOF)	TuneMix321	322.0481	153.8775	—	—	153.7	0.12	153.4(1)	0.31(1)
								153.5(6)	0.25(6)
	TuneMix621	622.0290	202.5721	—	—	203	0.21	202.8(6)	0.11(6)
								203(8)	0.21(8)
	TuneMix921	922.0098	243.5977	—	—	243.6	0.00	243.9(1)	0.12(1)
								243.5(6)	0.04(6)
	TuneMix1220	1221.9906	282.7998	—	—	282.2	0.21	283.1(1)	0.11(1)
								281.8(6)	0.35(6)
	TuneMix1520	1521.9715	316.6544	—	—	317	0.11	318.9(1)	0.70(1)
								316.5(6)	0.05(6)

*Note*: All adduct ions are [M+H]^+^. In ΔCCS_ref_ (%) columns: 100×|AutoCCS CCS - CCS_ref_|/CCS_ref_. ^1^CCS_Browser_ is obtained from the Agilent MassHunter IM-MS Browser 10.0 with raw .d files. ^2^CCS_PNNL_ is obtained from the PNNL CCS library (http://panomics.pnnl.gov/metabolites/) (Stow *et al.*, 2017; Zheng *et al.*, 2017). ^3^CCS_McLean_ is from the Unified CCS Compendium (https://mcleanresearchgroup.shinyapps.io/CCS-Compendium/, 2020/08/26 release) (Picache *et al.*, 2019) (#) in ^3^CCS_McLean_ and ^3^ΔCCS_McLean_ (%) columns represents the different data sources used in the CCS Compendium repository. For example, CCS values of (6) in the CCS_McLean_ were sourced from the interlaboratory experimental study (Stow *et al.*, 2017). For SLIM data, the quadratic function with polynomial calibration method was employed (Tune-Mix ion 321 was excluded due to low signal-to-noise). For Waters Synapt data, the linearized power function was used. For Bruker timsTOF data, the linear function was employed with IMS scan numbers used as substitute for drift time.

The Agilent IM-MS Browser is a user-friendly tool for visualization and processing of DTIMS data, which is the reference software for CCS calculation in the DTIMS scientific community. Here, we refer to the CCS determination using this vendor software as manual since it processes one file-compound-adduct at a time for stepped-field CCS calculations. In the case of single-field data, the user must manually select through the GUI each calibrant run and the corresponding runs to be calibrated. This user-interaction driven method is necessary for quality control and helps to educate users about the data but cannot be automated. Therefore, we developed AutoCCS using automated software components.

Inherent differences in feature detection algorithms between MZmine and IM-MS Browser led to small differences in CCS values. While the difference was minimal for most cases (under 0.2%), we observed a larger difference for a few ions using the stepped-field method. A detailed inspection revealed that the arrival time determined by the IM-MS Browser software when performing stepped-field CCS calculations could result in larger differences for low intensity ions due to two main factors: (i) the field used for the manual selection of the ion and (ii) the IM-MS Browser applies a Gaussian fit and MZmine uses the peak apex. While a Gaussian model is generally more accurate for determining the centroid of low intensity ions which are noisy, inconsistencies due to the manual selection of ions could be more detrimental. Overall, we observed that the differences are within acceptable ranges according to the instrument resolution.

### 3.2 Standard molecules with stepped-field DTIMS-MS

In addition to the tune-mix calibrants, we also evaluated AutoCCS for the automatic calculation of CCS using DTIMS-MS for well-studied metabolites and peptides (Supplementary Table S3). Previously collected raw data (Stow *et al.*, 2017; Zheng *et al.*, 2017) was re-processed for the three metabolites and seven peptides in positive and negative electrospray modes (only positive for D-Biotin). AutoCCS provided good reproducibility (0.4% RSD) and consistency against manually determined CCS values as well (Table 2). Once the target list of molecules with neutral masses was defined and the adducts in the AutoCCS configuration file were specified, AutoCCS automatically generated all adduct ions for each molecule, performed all calculations, generated various visualization plots and generated a single output table with the CCS and all the information of all molecules, including statistics such as mean and RSD per technical replicates. Using AutoCCS, all processing for metabolites took 17 min in an unattended fashion; in contrast, about 2 h were required to manually process the 15 raw .d files, including numerous mouse clicks as well as additional calculations in MS Excel to compare replicates.

**Table 2.** Results of CCS determination from stepped-field measurements in nitrogen drift gas using the demo dataset of the target compounds described in Supplementary Table S3

Name	Type	Adduct	Adduct *m/z*	CCS_avg_ (Å^2^)	%RSD	^1^CCS_Browser_ (Å^2^)	^1^ Δ CCS_Browser_ (%)	^2^CCS_PNNL_ (Å^2^)	^2^ Δ CCS_PNNL_ (%)
d-Biotin	Metabolite	[M+H]^+^	245.10	154.0411	0.40	154.0	0.03	154.16	0.08
		[M+Na]^+^	267.08	163.9419	0.63	164.3	0.24	164.45	0.31
Sucrose	Metabolite	[M+Na]^+^	365.11	174.7238	0.34	174.7	0.01	173.93	0.46
β-Nicotinamide adenine dinucleotide	Metabolite	[M+H]^+^	664.12	229.6815	0.41	229.3	0.18	229.46	0.10
		[M+Na]^+^	686.10	226.6511	0.05	226.1	0.24	226.24	0.18
		[M-H]^-^	662.10	230.1841	0.28	229.0	0.50	228.12	0.90
Angiotensin I	Peptide	[M+2H] ^2+^	648.85	386.06	0.42	386.2	0.04	386.13	0.02
		[M+3H] ^3+^	432.90	474.32	0.12	475.5	0.26	476.05	0.36
		[M+4H]^4+^	324.93	545.51	0.32	548.9	0.62	548.67	0.58
Angiotensin II	Peptide	[M+H]^+^	1046.54	312.73	0.89	313.4	0.22	314.2	0.47
		[M+2H]^2+^	523.78	355.88	0.51	354.1	0.49	354.43	0.41
		[M+3H]^3+^	349.52	434.70	0.04	435.3	0.14	435.63	0.21
Bradykinin	Peptide	[M+H]^+^	1060.57	315.35	1.10	312.9	0.79	312.97	0.76
		[M+2H]^2+^	530.79	343.14	0.53	343.1	0.02	343.47	0.10
		[M+3H]^3+^	354.20	449.70	0.12	447.7	0.44	448.16	0.34
Melittin	Peptide	[M+3H]^3+^	949.26	718.65	0.28	719.5	0.12	720.95	0.32
		[M+4H]^4+^	712.20	756.02	0.23	756.4	0.05	757.08	0.14
Neurotensin	Peptide	[M+2H]^2+^	836.96	433.72	0.39	434.5	0.18	434.89	0.27
		[M+3H]^3+^	558.31	524.65	0.26	525.1	0.09	525.54	0.17
Renin	Peptide	[M+3H]^3+^	586.98	519.66	0.12	519.0	0.12	519.58	0.02
		[M+4H]^4+^	440.49	634.50	0.70	635.9	0.21	636.34	0.29
Substance P	Peptide	[M+H]^+^	1347.74	357.83	1.11	359.3	0.40	359.66	0.51
		[M+2H]^2+^	674.37	399.02	0.15	398.4	0.16	398.87	0.04
		[M+3H]^3+^	449.92	497.19	0.20	496.9	0.05	497.36	0.03

*Note*: %RSD (Percent relative standard deviation) = 100 × standard deviation/CCS_avg_, In ΔCCS_ref_ (%) columns: 100×|AutoCCS CCS - CCS_ref_|/CCS_ref_. ^1^CCS_Browser_ is obtained from the Agilent MassHunter IM-MS Browser 10.0 with raw .d files. ^2^CCS_PNNL_ for metabolites is obtained from the PNNL CCS library (http://panomics.pnnl.gov/metabolites/) (Stow *et al.*, 2017; Zheng *et al.*, 2017) and ^2^CCS_PNNL_ for peptides is obtained from Supplementary Table S11 in Supplementary Material of the interlaboratory evaluation study paper (Stow *et al.*, 2017).

### 3.3 Single-field DTIMS-MS with RapidFire sample injection

We then demonstrated CCS calibration for data acquired from real-world metabolomic analyses of plant samples using SPE-IMS-MS with the single-field method and external calibration; stepped-field measurements are not feasible in many cases where analysis time is constrained due to either a front-end separation stage, limited sample amount or acquisition throughput needed.

All 7106 SPE-IMS-MS runs were processed in two weeks, which includes the computational time for preprocessing, feature finding and AutoCCS calculations, the latter of which required just 15 min on a desktop computer (3.6 GHz Intel Core i7, 16 GB 2400 MHz DDR4, Windows 10). Importantly, AutoCCS automatically assigned the corresponding tune-mix calibrant run based on the ionization mode (positive or negative) and the acquisition time stamp extracted from the raw file metadata information. On the other hand, manual data processing for CCS calibration and feature finding in this massive dataset would take more than a month due to many user interactions required.

When analyzing this data, we observed that unexpected fluctuations of pressure and temperature occurred during IMS data acquisition (Supplementary Fig. S7) and were sufficiently large to reduce CCS accuracy. Such large variations may happen due to instrumental issues (e.g. a malfunctioning flow controller or rough pump), an improper setting of the flow regime which are difficult to monitor in real time and correct during automated and large-scale studies.

**Fig. 2.**
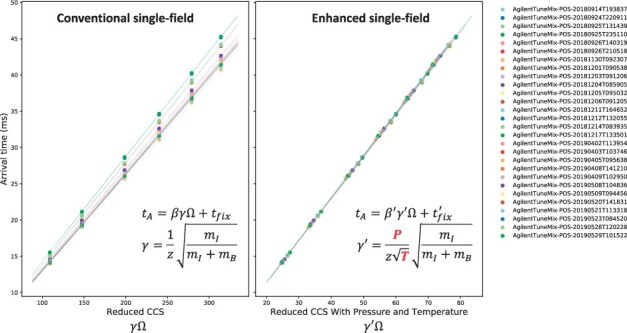
Single-field linear models for 28 tune-mix sample runs in positive electrospray mode using the same instrument as for SPE-IMS-MS analyses, conducted on 26 days from Sep. 2018 to May 2019 (see Section 2). Each dot represents a feature identified as a calibrant ion in Agilent tune-mix sample runs. Each line indicates the linear regressor from each tune-mix sample run. The plots show the impact of the enhanced single-field calibration, taking advantage of pressure and temperature when calibrating using multiple datasets for a set of long-term experiments

**Fig. 3.**
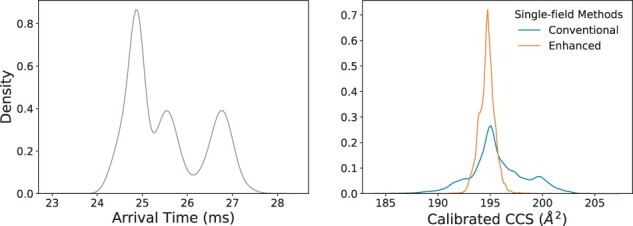
Distribution of replicate SPE-IMS-MS measurements by arrival time centroids and calibrated CCS values with conventional and enhanced single-field calibration of the internal standard rotenone [M+H]^+^ (*m*/*z* = 395.1489, ±20ppm). This internal standard ion was measured in 7106 runs, conducted on 26 days from September 2018 to May 2019 (see Section 2). The left panel shows three main distributions of arrival times due to parameter fluctuations across the multiple days. The right panel shows calibrated CCS values. Each SPE-IMS-MS run was calibrated using the linear regressor derived from the following and closest tune-mix run in the acquisition batch. With the same set of features, the proposed enhanced single-field method that takes into account the temperature and pressure from each run for regression, provided a small variance (194.7 with 0.4% RSD) and almost a single distribution in the calibrated CCS values for rotenone [M+H]^+^, while the conventional method provided a large variance (195.6 with 1.4% RSD)

We found that in cases where these fluctuations occur, the CCS error can be significantly reduced by taking into account the specific temperature and pressure values from each run as shown in Equation (3). Our enhanced single-field method incorporates the pressure and temperature well-recorded readings from each IMS-MS analysis, into the single-field CCS calibration (Fig. 2). Using this method, hourly fluctuations of temperature and pressure can be mitigated, beyond the daily fluctuations that are taken into account with the conventional single-field calibration. 7106 RapidFire runs were calibrated using the linear regressor derived from the following and subsequent closest tune-mix run in the acquisition batch. As shown in Figure 3, for protonated ions of the internal standard rotenone, the proposed enhanced single-field method provided a small variance (194.7 with 0.4% RSD) and almost a single distribution in the calibrated CCS values for rotenone ions despite an irregular distribution of arrival times due to temperature and pressure changes, while the conventional method provided a large variance (195.6 with 1.4% RSD) with the same set of features. This case demonstrates that the enhanced single-field method for DTIMS can correct any fluctuations due to changes in realized pressure and temperature, which could be encountered in real-world applications, and would be a benefit to the research community.

### 3.4 Calibration for SLIM IMS-MS (TAA, He)

We also conducted experiments to demonstrate the calibration methods for SLIM IMS-MS with measurements of TAA salts using helium as a buffer gas. The reference CCS values of the TAA used as external calibrant ions were obtained by the DTIMS stepped-field method. To compute the SLIM IMS-MS calibration curves, AutoCCS used the reference CCS values and the arrival times of the corresponding features detected by SLIM IMS-MS. The results of the two non-linear regression methods supported are shown in Supplementary Figure S8. High $R^{2}$ values of each calibration curve for the reference ions were observed for both modes, indicating that the two regression methods worked well and were comparable. Since IM-MS browser does not support non-linear calibration, we could not directly compare the CCS determination between the two software.

**Fig. 4.**
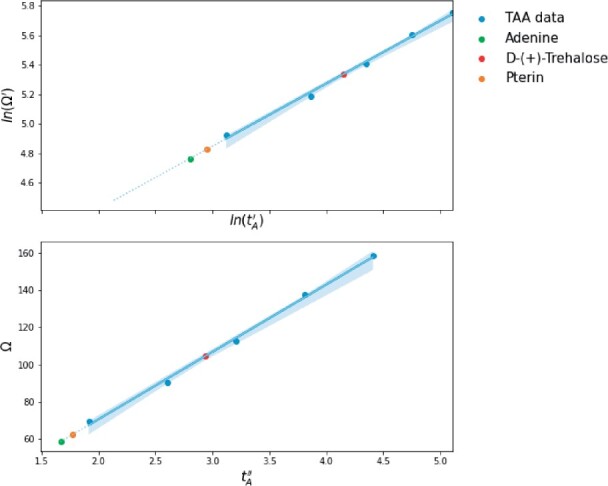
Calibrated results for TAA calibrants (blue dots) and three IROA standards in the linearized power function with 95% confidence interval (light blue). The solid line and dashed line represent the regression lines within the range of TAA data (i.e. interpolation) and outside of the range (extrapolation), respectively.

We demonstrated CCS determination for representative metabolites using three IROA standards measured by both DTIMS-MS and SLIM IMS-MS using helium gas. Table 3 shows that AutoCCS successfully calculates CCS values from measurements from both platforms. The linearized power regression provided more similar CCS values to DTIMS than the polynomial regression. However, the error for Pterin when using linearized power regression was notably higher at 2.26%. A possible explanation for this larger error could be that the measured arrival time of Pterin was slightly outside of the range of the TAA calibrants, and therefore CCS values calibrated in the extrapolation range would have a larger uncertainty level (Fig. 4). To improve the calibration, extrapolation uncertainty can be eliminated by measuring other calibrant ions in this range. The evaluation and optimization of different calibrants and experimental conditions is an active area of research for small molecules measured in SLIM systems.

**Table 3.** Results of CCS determination using AutoCCS for 3 IROA standards measured in helium gas via the DTIMS-MS and SLIM IMS-MS platforms

Compound name	Target adduct	Adduct *m*/*z*	^DT^CCS (Å^2^)	^SLIM^CCS_bin_ (Å^2^)	^SLIM^CCS_power_ (Å^2^)	Δ CCS_bin_ (%)	Δ CCS_power_ (%)
Adenine	[M+H]^+^	136.0624	58.44	63.53	58.51	8.71	0.12
d-(+)-Trehalose	[M+Na]^+^	365.1060	104.90	102.50	104.50	2.29	0.38
Pterin	[M+H]^+^	164.0574	63.85	65.90	62.41	3.21	2.26

*Note*: ^DT^CCS and ^SLIM^CCS represent the CCS values calculated by AutoCCS from DTIMS and SLIM IMS measurements, respectively. ^SLIM^CCS_bin_ and ^SLIM^CCS_power_ indicate the use of the two different regression methods in Supplementary Figure S8, a binomial regression and a linearized power regression, respectively. ΔCCS_reg_ (%) columns represent the CCS differences between DTIMS-MS and SLIM IMS-MS: 100×|^SLIM^CCS_reg_−^DT^CCS|/^DT^CCS (reg∈{bin, power}).

### 3.5 Limitations

As demonstrated in various case studies, the current version of AutoCCS can be directly used for CCS determination from experimental data measured in two instruments (Agilent DTIMS and SLIM) and substantially reduce time-consuming and labor-intensive tasks, especially for large-scale experiments. We also demonstrated that AutoCCS can be used with other commercial vendors, e.g. Waters Synapt and Bruker timsTOF. However, for Waters and Bruker data, conversions of the raw data into open format (e.g. mzML) and file formatting for IMS features are required since AutoCCS currently supports only Agilent MassProfiler cef and MZmine ouput csv format as input files.

Another limitation is that AutoCCS determines the CCS of an ion based on a peak apex or centroid rather than accounting for the width of the arrival time distribution; therefore, CCS distributions driven by ion structural ensembles cannot be characterized. For identifying CCS distributions of analyte ions in a stepped-field experiment, FWHMstep (Marchand *et al.*, 2017) can be utilized, which allows to characterize the analyte’s conformational diversity.

## 4 Conclusions

We present an open-source software, AutoCCS, for calculating the CCS of ions detected from various IMS platforms and data acquisition modes such as drift tube (Agilent), traveling wave (Waters Synapt and SLIM) and trapped (Bruker timsTOF) IMS-MS technologies. AutoCCS shows its effectiveness and utility to determine CCS values from various platforms, supporting different instruments, IMS-field methods, buffer gas (e.g. He or N_2_) and most importantly, an automatic workflow that generates reproducible results and is applicable for determining CCS in any omics study.

## Supplementary Material

btab429_Supplementary_Data
