# Supplementary material for: AutoCCS: automated collision cross-section calculation software for ion mobility spectrometry–mass spectrometry
Source: Bioinformatics. 2021 Jun 19;37(22):4193–201. doi: 10.1093/bioinformatics/btab429 (PMC9502155; doi:10.1093/bioinformatics/btab429)
Supplement: btab429_Supplementary_Data [file btab429_supplementary_data.docx]

Data and text mining

**Supplementary material for**

**AutoCCS: Automated Collision Cross Section calculation software for ion mobility spectrometry-mass spectrometry**

Joon-Yong Lee^1*†^, Aivett Bilbao^1*†^, Christopher R. Conant^1^, Kent J. Bloodsworth^1^, Daniel J Orton^1^, Mowei Zhou^1^, Jesse W. Wilson^1^, Xueyun Zheng^1^, Ian K. Webb^2^, Ailin Li^1^, Kim K. Hixson^1^, John C. Fjeldsted^3^, Yehia M. Ibrahim^1^, Samuel H. Payne^4^, Christer Jansson^1^, Richard D. Smith^1^, and Thomas O. Metz^1*^

^1^Earth and Biological Sciences Directorate, Pacific Northwest National Laboratory, Richland, WA, 99352, USA

^2^Department of Chemistry & Chemical Biology, Indiana University–Purdue University Indianapolis, Indianapolis, IN, 46202, USA

^3^Agilent Technologies, Santa Clara, CA, USA

^4^Department of Biology, Brigham Young University, Provo, UT, 84602, USA

*To whom correspondence should be addressed.

E-mail: [joonyong.lee@pnnl.gov](mailto:joonyong.lee@pnnl.gov), [aivett.bilbao@pnnl.gov](mailto:aivett.bilbao@pnnl.gov) and [thomas.metz@pnnl.gov](mailto:thomas.metz@pnnl.gov).

†These authors contributed equally to this work.

Demo dataset is publicly available at MassIVE (Dataset ID: MSV000085979).

- **Supplementary Tables**
- **Supplementary Figures**

**Table S1**. A list of parameters in a configuration file. ‘Single’ and ‘Step’ columns represent if each parameter is required in the ‘Single-field DT or TWIMS’ and ‘Stepped-field’ mode, respectively.

| Parameter | Description | Single | Step |
| --- | --- | --- | --- |
| mz_tolerance | Mass-to-charge tolerance (ppm) | O | O |
| suffix_raw | Suffix string for preprocessed raw data files. To handle the field numbers, add ‘{0}’ in the right position. E.g., _MA-d3-csum_{0}.mzML_c_dc_de. |  | O |
| suffix_meta | Suffix string for frame metadata files |  | O |
| frame_offset | Selected frame for each field by offsets to get metadata (e.g., field, pressure, and temperature). -1 indicates the use of the average values over all frames. | O | O |
| num_fields | Number of fields |  | O |
| drift_tube_length | Actual drift tube length (cm) |  | O |
| old_drift_tube_length | Drift tube length used in the acquisition software (cm) |  | O |
| neutral_mass | Neutral mass of the ion mobility buffer gas |  | O |
| substring_tunemix | Substring to discern the tune mix sample files | O |  |
| adducts | list of adduct ions to search |  | O |
| accumulation_time | Ion accumulation time (ms) in the SLIM-based IMS-MS | O |  |
| C | Enhanced duty cycle delay coefficient for power regression | O |  |

**Table S2**. A list of command-line options. ‘Single’ and ‘Step’ columns represent if each parameter is required in the ‘Single-field DT or TWIMS’ and ‘Stepped-field’ mode, respectively.

| Option | Description | Single | Step |
| --- | --- | --- | --- |
| --mode | CCS method mode limited to [single, multi]. single: single-field, multi: multi-field (stepped-field) mode | O | O |
| --feature_files | Feature files to determine CCS values | O | O |
| --framemeta_files | Frame meta files for samples | O | O |
| --output_dir | Directory to store output files | O | O |
| --config_file | Configuration file path | O | O |
| --ppm | Mass-to-charge tolerance (ppm). It will override mz_tolerance in config_params | O |  |
| --calibrant_file | Calibrant file that contains m/z and CCS values of reference calibrant ions | O |  |
| --calibration_curves | Calibration file that contains curves fit from tune mix samples | O |  |
| --calib_method | Calibration methods [‘poly’, ‘power’]. poly: using the polynomial function, power: using the linearized power function | O |  |
| --sample_meta | Meta file for samples | O |  |
| --colname_for_sample_type | Column name for sample types in ‘sample_meta’ | O |  |
| --colname_for_ionization | Column name for ionization modes in ‘sample_meta’ | O |  |
| --tunemix_sample_type | Sample type for tune mix samples in ‘sample_meta’ | O |  |
| --skip_calibrated_colname | Column name for the calibrated CCS values when you want to skip some files already having calibrated CCS values | O |  |
| --degree | Degree of the fitting polynomial for CCS calibration curves. It will be ignored if --calib_method = power. | O |  |
| --single_mode | Single method mode limited to [fit, batch]. fit: fitting calibration functions from calibrant runs, batch: not only the calibration but also the CCS determination. | O |  |
| --target_list_file | Target list file that contains the information (the exact mass and the identifier to link to the raw file) of target molecules |  | O |
| --output | Output file to store a table of CCS determinations for target molecules |  | O |
| --r2_threshold | Threshold value of $R^{2}$ |  | O |
| --num_isotopes_threshold | Threshold value of the number of isotopes |  | O |
| --intensity_rank_threshold | Threshold value of peak intensity ranks in a m/z window |  | O |
| --maxint | To select most intense peaks for CCS calculation |  | O |
| --format | Feature data file format ['cef','mzmine'] |  | O |

**Table S3.** An example of a target list file to apply in the stepped field mode. The “**CompoundID**” column must contain unique identifiers to distinguish from other compounds, which are feasible for a valid filename. The “**CompoundName**” column has the compound names or short descriptions and the “**ExactMass**” column contains the monoisotopic mass of the neutral target molecule to be used to compute m/z values of the adduct ions. In the “**UniqueID4DfileNames**” column, users must provide the unique text strings included in the corresponding file names. Otherwise, the files cannot be found for target compounds.

| **CompoundID** | **CompoundName** | **Exact Mass** | **UniqueID4DfileNames** |
| --- | --- | --- | --- |
| S0000001 | D-Biotin | 244.0882 | 58-85-5 |
| S0000002 | Sucrose | 342.1162 | 57-50-1 |
| S0000003 | beta-Nicotinamide Adenine Dinucleotide | 663.1091 | 53-84-9 |
| Substance_P | Substance P | 1346.7281 | Metabolite_Mix_7 |
| Bradykinin | Bradykinin | 1059.5614 | Metabolite_Mix_7 |
| Neurotensin | Neurotensin | 1671.9096 | Metabolite_Mix_7 |
| Fibrinopeptide_A | Fibrinopeptide A | 1535.6852 | Metabolite_Mix_7 |
| Melittin | Melittin | 2844.7541 | Metabolite_Mix_7 |
| Angio_I | Angio I | 1295.6775 | Metabolite_Mix_7 |

**Table S4.** The column names of a CSV output file including CCS results in AutoCCS.

| **Column Names** | **Descriptions** |
| --- | --- |
| Compound_id | Compound ID, the same to CompoundID in the target list file. |
| name | Compound name, the same to CompoundName in the target list file |
| Ionization | Ionization of the adduct ion |
| adduct | Adduct ion |
| adduct_mz | mass for the adduct form |
| ccs_avg | an average of CCS values of same adducts for all the replicates |
| ccs_rsd | a relative stdv of CCS values of same adducts for all the replicates |
| ccs | a CCS value of this adduct for this replicate |
| dt_# | Arrival time of a corresponding feature in #th field |
| intensity_org_# | original intensity of a corresponding feature in #th field |
| intensity_z_# | z-score of an intensity of a corresponding feature in #th field |
| mass_error_# | mass error of a corresponding feature in #th field (ppm) |
| num_features | number of features found in all step fields |
| intercept | intercept of a CCS regression line |
| slope | slope of a CCS regression line |
| k0 | K0 computed from a CCS regression line |
| p_value | p value of a CCS regression line |
| r2 | R^2^ value of a CCS regression line |
| replicate | original file for this CCS computation (e.g., .d file) |

**Table S5**. An example of calibrant file using the Agilent tune mix ions to demonstrate the single field method.

| **CCS** | ***m/z*** | **Exact Mass** | **Ionization** | **z** |
| --- | --- | --- | --- | --- |
| 121.3 | 118.0863 | 117.07836 | pos | 1 |
| 153.7 | 322.0481 | 321.04016 | pos | 1 |
| 203 | 622.029 | 621.02106 | pos | 1 |
| 243.6 | 922.0098 | 921.00186 | pos | 1 |
| 282.2 | 1221.9906 | 1220.98266 | pos | 1 |
| 317 | 1521.9715 | 1520.96356 | pos | 1 |
| 351.3 | 1821.9523 | 1820.94436 | pos | 1 |
| 383 | 2121.9332 | 2120.92526 | pos | 1 |
| 413 | 2421.914 | 2420.90606 | pos | 1 |
| 441.2 | 2721.8948 | 2720.88686 | pos | 1 |
| 140 | 301.9981 | 303.00604 | neg | 1 |
| 180.8 | 601.979 | 602.98694 | neg | 1 |
| 255.3 | 1033.9881 | 1034.99604 | neg | 1 |
| 284.8 | 1333.9689 | 1334.97684 | neg | 1 |
| 319 | 1633.9498 | 1634.95774 | neg | 1 |
| 352.6 | 1933.9306 | 1934.93854 | neg | 1 |
| 380.7 | 2233.9115 | 2234.91944 | neg | 1 |
| 413 | 2533.8923 | 2534.90024 | neg | 1 |
| 432.6 | 2833.8731 | 2834.88104 | neg | 1 |

**Figure S1.** An example configuration file in the stepped field mode. Refer to **Table S1** for detailed configuration parameters.


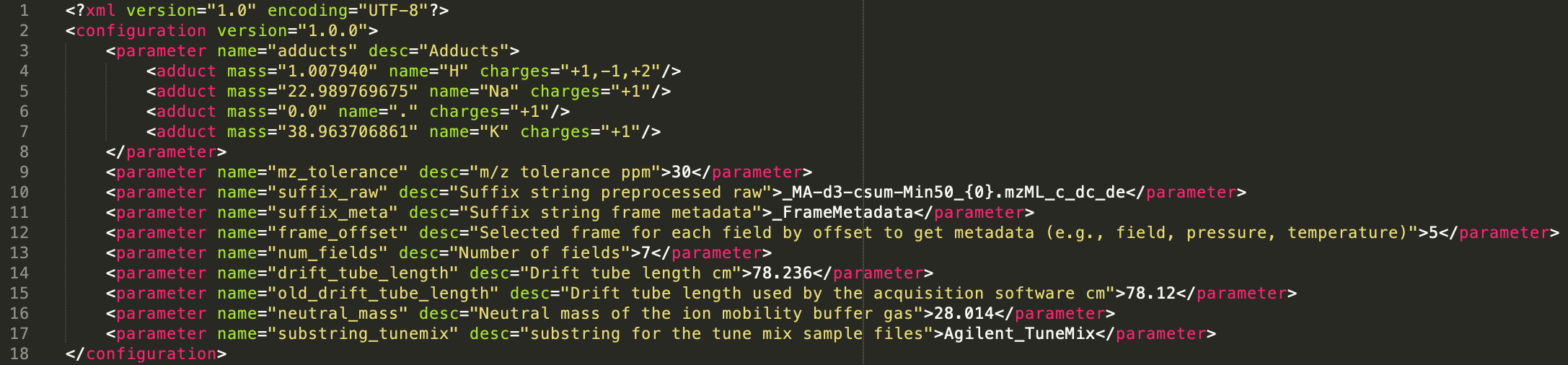


**Figure S2.** An example plot of the CCS regression line in the stepped field method. Each dot indicates the selected features within the pre-defined *m/z* tolerance. The size of dot represents the peak intensity. The red line shows the linear regression fit to the features of each field. The title represents the original feature file in which this computation performs and some detailed information such as the adduct ion, its *m/z*, mass error, z-scores of intensity values, the CCS value, and its R^2^ is described in the plot. This file was automatically generated from AutoCCS: S0000006_pos_[M+H].pdf. This CCS determination shows <0.03% difference from the reference CCS value (243.6 Å^2^) for the TuneMix921 (**Table 1**). If there are multiple replicate files for a target compound, these should be added in new rows. Please see other <compound_id>_<ionization> _<adduct>.pdf files.

**Figure S3.** Example plots of the intensity distributions of all features and the selected features in this distribution for TuneMix921 (**Table S1**). Each box indicates a different field and from top to bottom shows the lowest to the highest voltage fields, respectively. In each box, the x and y axes indicate the log-scale of original intensities and kernel density estimation (KDE) of its normalized distribution, respectively. Each dot represents the selected features within a ppm tolerance (according to mz_tolerance setting in the configuration xml). Blue and green dots indicate the selected features for [M+H] and [M+K], respectively. If there are multiple replicate files for a target compound, these should be added in new columns. Please see other <compound_id>_<ionization> _intensity_dist.pdf files.

**Figure S4.** Example plots of the frame meta data to show the change of temperatures, pressures, and field voltages of S0000001 (**Table S4**). There are three replicates and the experimental values for CCS-Pos-1b are different from the other two, likely due to the relative time between measurements. Each dot indicates the selected frame for each field by offsets to obtain metadata according to frame_offset in the configuration file.

**Figure S5.** Calibration regressors of tune-mix ions generated from (a) RapidFire-IMS-MS (linear) and (b) SLIM-based IMS-MS (polynomial).

**Figure S6.** Software tools and file structure with input/output folders used in AutoCCS. 1) The PNNL-PreProcessor was used to generate new raw files (MassHunter “.d” or UIMF format) with all frames (ion mobility separations) summed into a single frame, apply smoothing in the ion mobility dimension and export frame metadata text files containing the IMS acquisition conditions (e.g., electric fields, pressure, and temperature). 2) Proteowizard MSConvert was used for converting the raw single-frame files to mzML files. The arrival time was parsed as a substitute for retention time to generate “LC-MS-like” files using an R script. 3) MZmine 2 was used to perform feature finding and generate a csv list of features found for each mzML file. 4) AutoCCS was used to calculate the CCS. For single-field and SLIM data, new csv lists of features were generated in the “V_Results” folder, adding a column with the CCS values. For stepped-field data, in the “V_Results” folder various visualization plots are generated in pdf files for each target molecule and a single output table with the CCS and all the information of all target molecules. Configuration files specific for each experiment can be found in the accompanying data shared in MassIVE (Accession: MSV000085979).

**Figure S7.** Daily fluctuations of pressure and temperature during data acquisition for plant metabolomics samples. Large fluctuations in the pressure were observed during December 2018.


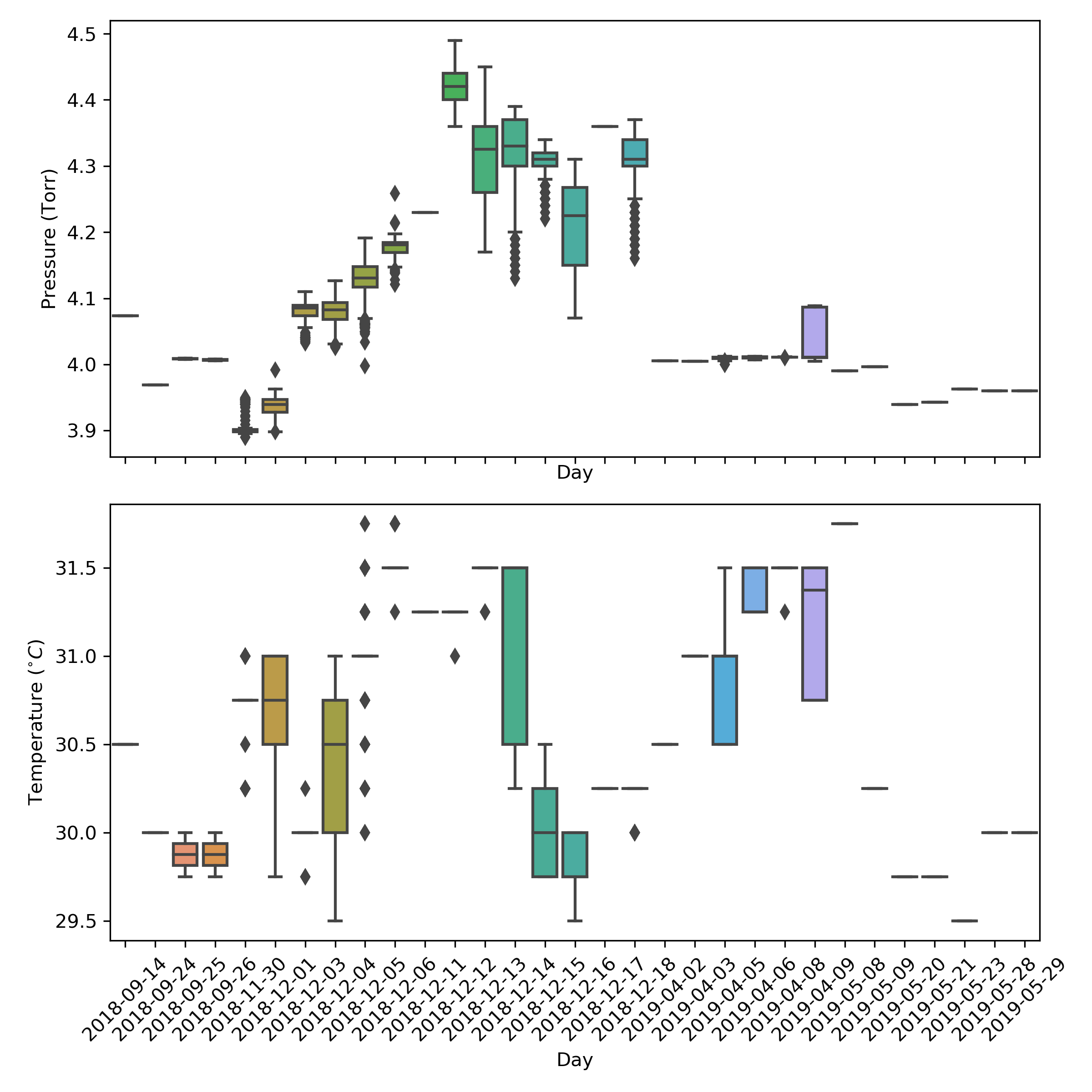


**Figure S8.** SLIM IMS-MS calibration curves built from the TAA calibrants using two different regression methods in AutoCCS: (a) binomial regression and (b) linearized power regression. Different colors represent each of the four replicate runs.
